# Modification Strategies of Kapok Fiber Composites and Its Application in the Adsorption of Heavy Metal Ions and Dyes from Aqueous Solutions: A Systematic Review

**DOI:** 10.3390/ijerph19052703

**Published:** 2022-02-25

**Authors:** Cybelle Morales Futalan, Angelo Earvin S. Choi, Hannah Georgia O. Soriano, Melbourne Klein B. Cabacungan, Jeremiah C. Millare

**Affiliations:** 1 Department of Community and Environmental Resource Planning, University of the Philippines, Los Baños 4031, Laguna, Philippines; 2 Department of Chemical Engineering, De La Salle University, Taft Avenue, Manila 2401, Metro Manila, Philippines; angelo.choi@dlsu.edu.ph; 3 School of Chemical, Biological, and Materials Engineering and Sciences, Mapua University, 658 Muralla St, Intramuros, Manila 1002, Metro Manila, Philippines; hgosoriano@mymail.mapua.edu.ph (H.G.O.S.); mkbcabacungan@mymail.mapua.edu.ph (M.K.B.C.); jcmillare@mapua.edu.ph (J.C.M.)

**Keywords:** kapok fiber, bioadsorbents, pre-treatment, surface modification, composites, adsorption

## Abstract

Kapok fiber (*Ceiba pentandra*) belongs to a group of natural fibers that are mainly composed of cellulose, lignin, pectin, and small traces of inorganic compounds. These fibers are lightweight with hollow tubular structure that is easy to process and abundant in nature. Currently, kapok fibers are used in industry as filling material for beddings, upholstery, soft toys, and nonwoven materials. However, kapok fiber has also a potential application in the adsorptive removal of heavy metal ions and dyes from aqueous systems. This study aims to provide a comprehensive review about the recent developments on kapok fiber composites including its chemical properties, wettability, and surface morphology. Effective and innovative kapok fiber composites are analyzed with the help of characterization tools such as scanning electron microscopy, X-ray diffraction, X-ray photoelectron spectroscopy, thermogravimetric analysis, Fourier transform infrared spectroscopy, energy-dispersive X-ray spectroscopy, and Brunauer-Emmett-Teller analysis. Different pre-treatment methods such as alkali and acid pre-treatment, oxidation pre-treatment, and Fenton reaction are discussed. These techniques are applied to enhance the hydrophilicity and to generate rougher fiber surfaces. Moreover, surface modification and synthesis of kapok fiber-based composites and its environmental applications are examined. There are various methods in the fabrication of kapok fiber composites that include chemical modification and polymerization. These procedures allow the kapok fiber composites to have higher adsorption capacities for selective heavy metal and dye removal.

## 1. Introduction

Heavy metal ion and dye pollution caused by industrialization and urbanization has caused significant hazards to both environmental and human health [1,2]. Industries release untreated wastes that contain both organic and inorganic pollutants. These range from metal salts to synthetic organic chemicals [3,4]. Large amounts of heavy metal polluted wastewaters contain a significant amount of Hg, Cu, Cd, Pb, and Zn [5] generated by industrial wastewater discharges from alloy industries, fertilizer plants, battery manufacturing, smelting, paint pigments, semiconductor processing plant, and electroplating plant [6]. The long-term exposure of humans to high concentrations of Hg, Cu, Pb, Cd, and Zn is known to cause several health concerns including major damage to the circulation, immune and reproductive system, liver failure, diabetes, mucosal irritation, kidney failure, and irritation of the central nervous system [7,8,9,10,11]. The United States Environmental Protection Agency (US EPA) has set the maximum daily intake of 0.004, 0.001, 0.0001 and 0.04 mg/kg of Pb, Cd, Hg and Cu, respectively [12]. In drinking water, US EPA has set the maximum contaminant level for Hg, Cd and Cu at 0.03 µg/L [13], 5.0 µg/L [14], and 1.3 mg/L [15], respectively. The maximum Pb content in drinking water is set at <10 ppb by WHO [16]. Hence, it is important to remove Hg, Cu, Pb, Cd, and Zn from wastewater before its release into any body of water.

Dyes are classified as either synthetic or natural chemical substances that are used to provide color to numerous substrates such as waxes, leather, fur, plastics, cosmetics, greases, and textile materials [4,17]. Because of their complex and large molecular structures, dye molecules remain in bodies of water for a long time, resisting degradation by natural mechanisms such as microbial interactions and sunlight [18]. These colored effluents restrict the amount of sunlight that penetrate the affected bodies of water, lowering the photosynthetic activity of aquatic flora and, as a result, influence an aquatic ecosystem’s overall food source. Cationic dyes such as methyl orange (MO) and methyl blue (MB) are carcinogenic, teratogenic, and mutagenic, all of which is dangerous for health. Exposure to MO and MB can cause severe illnesses and diseases such as skin irritation, dyspnoea, dermatitis, eye burn, and diarrhea [19]. Dyes are widely used in textile industries and are a major contributor to colored-wastewater production with high chemical oxygen demand (COD) concentration [20,21]. Printing and textile industries also produce anionic dyes including Orange G and Orange II that are considered to be teratogenic and carcinogenic in nature [22,23]. The ingestion of dye at a high concentration has numerous detrimental effects on human health such as allergies, asthma, failure of the reproductive system, brain, liver and kidneys [24]. The release of wastewaters with high concentration of COD will result in anaerobic conditions and depletion of oxygen in water bodies. The Taiwan Environmental Protection Agency has set the maximum contaminant level of COD to 100 mg/L. Meanwhile, the Philippine Department of Environment and Natural Resources has set the standard of COD at 30 to 60 mg/L in freshwater and marine water bodies, respectively [25].

Various methods have been developed to remove heavy metals and dyes from aqueous systems. These methods include membrane filtration, chemical coagulation and flocculation, biological treatment, ion exchange, photocatalytic degradation, and adsorption. Most of these methods are found have a high financial cost, high operational costs, and require a complex setup [26]. Adsorption is preferred over other methods due to its design flexibility, high-quality treated effluents, and the capacity to regenerate adsorbents [27]. Other advantages of adsorption include its simplicity of operation, provide relatively high efficiency, and ability to remove a wide spectrum of organic and inorganic pollutants, even at extremely low levels [28].

Adsorption is a surface process that is common in the removal of organic and inorganic pollutants. This phenomenon proceeds when a solution that contains a solute meets a surface structure, at which point intermolecular forces of attraction cause the solute to be deposited or concentrated on the solid surface [27]. According to the study of Kobya et al. [29], the principal factors that directly affect adsorption are adsorbent porosity and surface area, adsorbent weight, temperature, initial solute concentration, pH, and the mechanism of adsorption. Bioadsorbents and commercial bio-adsorbents are two types of adsorbents used to effectively remove heavy metal ions and dyes. When compared to commercial adsorbents, bioadsorbents are made from biological materials that are inexpensive. Commercial adsorbents are materials that are manufactured in large quantities. Activated carbon, alumina, silica gel, graphene, and magnetic adsorbents are all mass produced [27]. A cost analysis is an important factor to be considered when proceeding with a wastewater project. Since the total cost of the project is determined by the cost of the adsorbent [27], a low-cost option that has almost same function will be preferred.

Recently, researchers around the world have been taking interest in the treatment capabilities of kapok as an adsorbent. Kapok fiber (KF) is a silky fiber that encloses the seeds of kapok trees (Ceiba pentandra). It has a yellowish or light brown color with a silk-like luster. In contrast to cotton, KF is composed of single-celled plant hairs that are lignified and not attached to the seed grains [30]. Kapok trees belong to the family of *Bombaceae* and are commonly found in Asia, Africa, and South America. KF and its composite materials have been gaining attention due to their unique features and have been utilized in various applications [30]. Lignocellulosic materials in plants have been evaluated for their effectiveness in the sorption of heavy metal ions and even nonviable plant biomass have shown to effectively bind toxic metals [31]. KF is a promising biomaterial for use in the adsorption of heavy metals because of its hollow, microtubular structure [32]. However, the wax layer on the surface inhibits the access of hydrophilic coloring agents and heavy metal-containing solutions to the surface of KF [33]. To alter its intrinsic properties and surface characteristics, pre-treatment is required. It also cleans the fiber surface, making it subsequently easier for any bonding agent to attach. Several pre-treatment and modification methods have been determined to modify surface properties via removal of surface wax and lignin that would render KF to become hydrophilic. Chemical treatments such as alkali treatments and acid treatments are used to remove pectin, lignin, natural oils, and wax that envelope the fiber on its outer surface. Oxidation treatments modify KF by removing the phenolic compounds that change its property from hydrophobic to hydrophilic [33].

In the present work, different methods of pre-treatment and surface modification of KF that transform the wettability from a hydrophobic to a hydrophilic surface are presented. The specific objectives of the study are as follows: (1) to provide an in-depth review of the various pre-treatment methods and surface modifications applied to KF; (2) to evaluate the structure and properties of modified KF from various synthetic routes; and (3) to analyze of the adsorptive performance of the KF composites.

## 2. Kapok Fibers

Kapok (*Ceiba pentandra* L.) is a natural and cellulosic fiber that contains cellulose, hemicellulose, lignin, pectin, and wax [34]. It is composed of two layers, namely, of cellulose microfibrils (outer layer) and fiber axis (internal layer) [35]. These fibers are cylindrical, yellowish to brown in color, lightweight [36], abundant in nature, biodegradable, and easy to process [37]. Kapok fiber is most commonly used commercially in the textile industry as nonwovens, fillings for soft toys, beddings, upholstery, and sleeping bags [38,39,40]. Due to its oil-adsorption properties, KF is also used as a wicking material for mechanical lubricants [41]. Some water-safety equipment such as life preservers is also made of KF because of its buoyancy properties [42].

### 2.1. Structure and Properties

**Figure 1:**
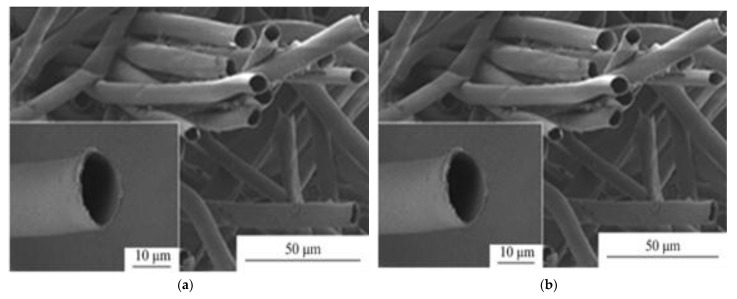
SEM image of (**a**) kapok fibers [47]. Reprinted by permission from Springer Nature Customer Service Centre GmbH: Springer Nature, Chinese Journal of Polymer Science, Investigation on sound absorption properties of kapok fibers, Xiang, H.F., Wang, D., Liua, H.C., Zhao, N., & Xu, J., Copyright (2013). SEM image of (**b**) cross section of kapok fibers [46]. Reprinted from Industrial Crops and Products, 61, Dong, T., Wang, F., & Xu, G., Theoretical and experimental study on the oil sorption behavior of kapok assemblies, 325–330, Copyright (2014), with permission from Elsevier.

**Figure 2:**
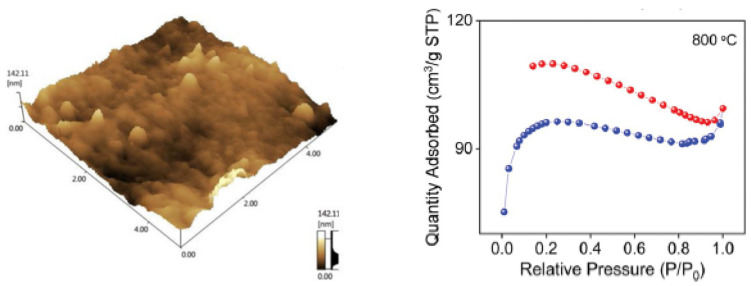
AFM image of raw KF (**left**) [48]. Reprinted by permission from Springer Nature Customer Service Centre GmbH: Springer Nature, Chinese Journal of Polymer Science, Investigation on sound absorption properties of kapok fibers, Xiang, H., Wang, D., Liua, H., Zhao, N., Xu, J., Copyright (2013). Nitrogen adsorption and desorption isotherm of kapok fibers pyrolyzed at 800 °C (**right**) [49]. Reprinted (adapted) with permission from Song, P.; Cui, J.; Di, J.; Liu, D.; Xu, M.; Tang, B.; Zeng, Q.; Xiong, J.; Wang, C.; He, Q.; et al., Carbon Microtube Aerogel Derived from Kapok Fiber: An Efficient and Recyclable Sorbent for Oils and Organic Solvents. ACS Nano, 14, 595–602, Copyright 2020 American Chemical Society.

The fiber, per pod, weighs 12–15 g and each strand measures 10–35 mm in length [38]. Furthermore, it has a specific density of 1.31 g/cm^3^ [43]. KF has a diameter of 20–43 µm with 1–3 µm wall thickness [38]. It is important to identify the fiber diameter since it is a factor of fluid flux along the surfaces of the KF [44,45]. As seen in Figure 1, SEM images show a fine hollow tube shape with a large lumen [30,46] and a smooth surface due to the presence of a thick layer of wax [1,47]. AFM topographic image of KF is displayed in Figure 2. The surface roughness of KF ranges from 140 to 155 nm [48]. Brunauer-Emmett-Teller (BET) analysis (Figure 2) shows the N_2_ adsorption–desorption isotherm of KF that has been pyrolyzed at 800 °C with surface area of 324 m^2^/g [49]. Lim and Huang [50] investigated the wettability of KF with water and diesel. Their results show a larger contact angle of 117° for water, while diesel exhibited a smaller contact angle at 13°. Contact angles in water, ethanol, and hexane have also been reported to be 120°, 12°, and 13°, respectively [51]. KF has hydrophobic properties due to the waxy layer on the surface [52], limiting its ability to remove hydrophilic dyes and heavy metals [33]. To improve the wettability and adsorbing capabilities of KF, surface modification and pre-treatment are recommended. Since adsorption processes occur in aqueous solutions, a hydrophilic surface is desirable in heavy metal and dye removal. The contact between the adsorbent and the contaminant in the aqueous solution is a factor in overall adsorption capacity, as a higher contact area between the pollutants and the KF surface means more adsorption capacity. Thus, KF is rarely used as an adsorbent material to remove contaminants from an aqueous solution [53]. The surface topography of KF also affects the adsorption capacity. Heavy metals and dyes can effectively attach to rougher surfaces due to higher surface area exposed and more empty spaces (binding sites) available for adsorption [39,44].

### 2.2. Composition of Kapok Fibers

**Figure 3:**
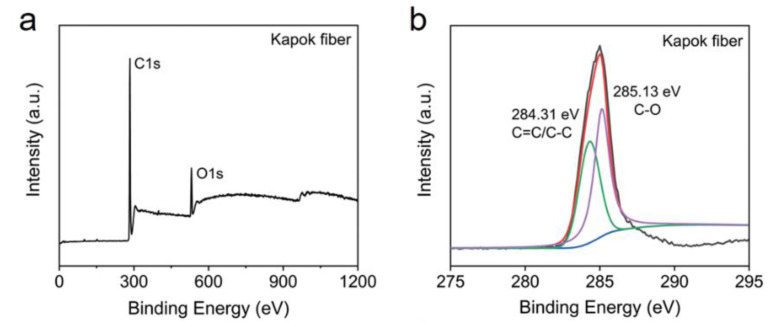
(**a**) XPS spectra of kapok fiber and (**b**) XPS region of kapok fiber that is related to C1s [49]. Reprinted (adapted) with permission from Song, P.; Cui, J.; Di, J.; Liu, D.; Xu, M.; Tang, B.; Zeng, Q.; Xiong, J.; Wang, C.; He, Q.; et al., Carbon Microtube Aerogel Derived from Kapok Fiber: An Efficient and Recyclable Sorbent for Oils and Organic Solvents. ACS Nano, 14, 595–602, Copyright 2020 American Chemical Society.

KF contains cellulose, hemicellulose, lignin, and pectin with low amounts of inorganic substances [38,43]. Figure 3 shows the XPS (X-ray photo electron spectroscopy) results where KF is mostly composed of C (carbon) 1s and O (oxygen) 1s with peaks around ~285 eV [54] and ~530 eV [55], respectively. Song et al. [49] reported that C 1s is also linked to C = C/C-C (284.31 eV) and C-O (285.13 eV) bonds. Apart from carbon and oxygen, Yang et al. [56] and Cao et al. [57] identified a soft peak (400 eV) between C 1s and O 1s as N (nitrogen) 1s. Given that adsorption is primarily based on the interaction between the adsorbate and binding sites found on the KF surface, XPS is a useful characterization approach. The parameters required for successful KF adsorption can be adjusted and varied by knowing the chemical composition [58].

X-ray diffraction (XRD) patterns provide the crystalline structures of KF, where peaks ranging from 10° to 30° are due to lignin, amorphous cellulose, and hemicellulose [57]. The crystallinity index of KF ranges from 20–40%, depending on the surface modifications performed [51,59]. Natural KF has crystallographic planes (101) and (002) and two visible peaks around 2θ = 15° and 2θ = 22°, which correspond to the presence of lignin and cellulose, respectively [60]. These factors help to identify areas that are more amorphous. These amorphous regions are known to involve high energy adsorption sites, since it is densely packed with defects and functional groups [61].

**Figure 4:**
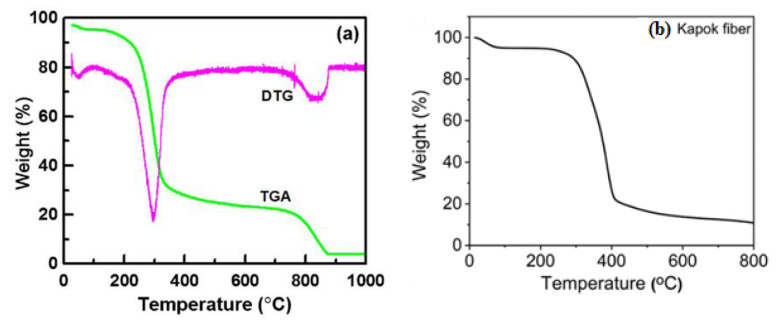
(**a**) TGA and DTG curves of KF [60]. Reprinted from Journal of Material Science & Technology, 34, Wang, J.-R., Wan, F., Lü, Q.-F., Chen, F., & Lin, Q., Self-nitrogen-doped porous biochar derived from kapok (Ceibainsignis) fibers: Effect of pyrolysis temperature and high electrochemical performance, 1959–1968, Copyright (2018), with permission from Elsevier. (**b**) TGA curve of KF [49]. Reprinted (adapted) with permission from Song, P.; Cui, J.; Di, J.; Liu, D.; Xu, M.; Tang, B.; Zeng, Q.; Xiong, J.; Wang, C.; He, Q.; et al., Carbon Microtube Aerogel Derived from Kapok Fiber: An Efficient and Recyclable Sorbent for Oils and Organic Solvents. ACS Nano, 14, 595–602, Copyright 2020 American Chemical Society.

The thermal stability of the fibers can be assessed using a thermogravimetric analysis (TGA). In chemisorption, where bonds are formed, increasing the temperature increases the rate of chemical reactions. The temperature increase will also modify the chemistry of the adsorbent and adsorption sites. Greater adsorption at higher temperatures may also imply that the chemical adsorption process is endothermic [62]. As shown in Figure 4, thermal decomposition measured for KF under an inert atmosphere is around 83% weight loss that occurs in a range of 250–900 °C [57]. Wang et al. [60] investigated the thermal degradation of KF using derivative thermogravimetry (DTG) and TGA, wherein a small decrease in the weight was observed below 100 °C, found to be caused by water molecule evaporation. Then, a decrease of approximately 10% in weight of KF was noted at temperatures between 100–230 °C, due to hemicellulose decomposition [60,63]. As a result of cellulose decomposition and the rearrangement of macromolecules present in KF, a large decrease in weight to 63% was observed at 230–380 °C up to 770 °C [60,64]. Moreover, the loss of moisture contributed to a 5% weight decrease at temperatures between 20–78 °C [49,65]. The weight of KF decreased to 77% at 235–405 °C because of the degradation of cellulose and lignin, with only 11% of the original weight remaining when it reached 800 °C [49,66].

Fourier transform infrared (FTIR) results of raw KF show different functional groups that are present in the fiber. The band at 3404 cm^−1^ is associated with hydroxyl stretching from plant wax, fatty acids and water [52,67,68]. Cellulosic components CH_2_ and CH_3_ are found at band 2917 cm^−1^ [67,69]. C=O (carbonyl stretching) is determined at 1740 cm^−1^ and 1374 cm^−1^, which refers to the presence of hemicellulose [66,67]. Aromatic C=C was also observed at peaks from 1595 cm^−1^ to 1425 cm^−1^, which indicates the presence of lignin [66,67]. The hydrophobicity of KF is due to the presence of hemicellulose and lignin. The removal of these groups is critical in the adsorption process as it prevents the aqueous solution from contacting the fiber surface [58].

### 2.3. Adsorption Performance of Raw KF

The adsorption performance of raw kapok fibers for the removal of lead and mercury was examined and observed by Wang et al. [70] and Yang et al. [56]. The adsorption capacity of the untreated KF was found to be 4.70 mg/g and 39.9 mg/g for the removal of Pb and Hg, respectively. At a peak 2917 cm^−1^ the stretching of CH_2_ and CH_3_ reflect the presence of plant wax, which implies the natural hydrophobicity of the raw KF. It was also observed during the experimentation stages that the raw KF remained afloat at the surface of the solution during the adsorption process. However, due to the abundance of hydroxyl found in KF, it was still able to capture metal ions on its surface. Adsorption using an untreated KF can be used as a basis to compare the adsorption performance of other treated and modified KF [56,70].

## 3. Pre-Treatments and Surface Modification of Kapok Fibers

The fabrication of a KF composite involves the addition of xerogels and aerogels, clays, polymers, or nanomaterials [49]. However, these have poor compatibility because of hydrophobicity, low adhesion, and low adsorption capacity of KF [48,49]. KF and its composites can be used as an adsorption material for heavy metal ions and dyes in an aqueous solution with the right pre-treatment and surface modifications [33,43,59].

Some of the known applications include the sorption of oil from fibers [10,46], adsorption of heavy metal and dyes using KF [68,71], and the fabrication and modification of KF for the removal of heavy metal ions and dyes [1,8,52,56]. Pre-treatment of KF from previous studies includes acid/alkaline treatment [70], oxidation treatment [72], and Fenton reaction [73]. Additionally, previous studies in the literature discuss different surface modifications on KF, such as coating [74] and blending [75].

KF is pretreated to alter its surface features and intrinsic properties [33], so as to improve the adsorption qualities of KF and the bonding agent, and to clean the fiber surface of any impurity [76]. Surface modification is also applied to reverse the wetting properties of KF [68]. Moreover, the surface modification of KF also prevents damages to its thin structure by treating it with other additives to increase its mechanical properties [77]. The wettability, adhesion characteristics, and biocompatibility of KF are typically poor, requiring modification prior to application or any additional processing such as coating or blending with functional materials [74].

Chemical pre-treatments are expensive due to the chemical agents utilized [78]. Alkaline pre-treatment is a widely used technique for altering the cellulose structure, allowing chemicals to penetrate the fiber more easily [79]. The main disadvantages of acid pre-treatment include the formation of by-products at strong acidic conditions, increased degradation of complex substrates, and high costs of acids used for the pre-treatment [80]. Meanwhile, Fenton reaction treatment is easy to prepare and operate [81] only if carried out on the laboratory scale. Industrial application is limited due to the harsh synthetic conditions, complex synthesis, expensive catalyst costs, and complicated reactor design [81]. Pre-treatment with oxidation is a faster and effective approach, but it yields hazardous chemicals such as organochlorine compounds. Oxygen and catalysts are used for surface modification that can improve the fiber-matrix interfacial bonding, surface morphology, and wettability by forming strong chemical bonds. In addition to removing impurities, this procedure increases the number of reaction sites. However, some surface modification procedures take time. Additionally, polymerization alone is a complicated procedure that requires around twelve (12) hours to complete, and consumes a lot of reagents [82,83].

### 3.1. Pretreatments of Kapok Fibers

#### 3.1.1. Acid/Alkaline Pretreatment of Kapok Fiber

**Figure 5:**
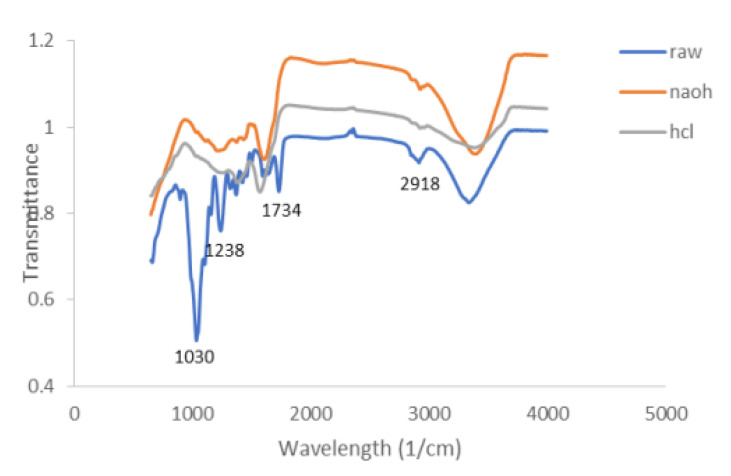
FTIR graph of raw, NaOH-treated, and HCl-treated KF [65].

Acid and alkali pretreatment improves surface topography, solubility properties, and reactivity of KF by degrading cellulose, pectin, natural oils, and lignin in fibers [69,84,85]. Sulfuric, hydrochloric, nitric, acetic, or maleic acid can be used to pre-treat fibers in diluted or concentrated forms [86]. Alkaline pre-treatment on fibers, on the other hand, involves a concentrated aqueous solution with a strong base [87] such as sodium hydroxide, potassium hydroxide, or calcium oxide. Abdullah et al. [65] pretreated KF with HCl and NaOH to remove wax, lignin, and impurities. The FTIR (Figure 5) of treated KF showed a higher broad absorption band at 3410 cm^−1^ than raw KF [65]. This trend implied that hydroxyl groups from the pretreatment combined effectively with the cellulose and reduction of plant wax in KF took place [88]. The NaOH-treated KF exhibited decreasing intensity at bands 1740 cm^−1^ and 1245 cm^−1^. This implies the removal of carboxylic group (oil and fatty acid) present in KF through alkalinization [65,69]. TGA was performed on the untreated, acid, and alkali pre-treated KF, whereby all samples displayed an initial weight loss of 1–2 mg from 39 °C to 108 °C due to the evaporation of moisture and volatile compounds [65,89]. Raw KF recorded around 2–3.5 mg weight loss that occurred between 237 °C and 367 °C because of dehydration and breaking down of cellulose and lignin. The complete degradation of cellulose and lignin was observed at 358–750 °C [65].

**Figure 6:**
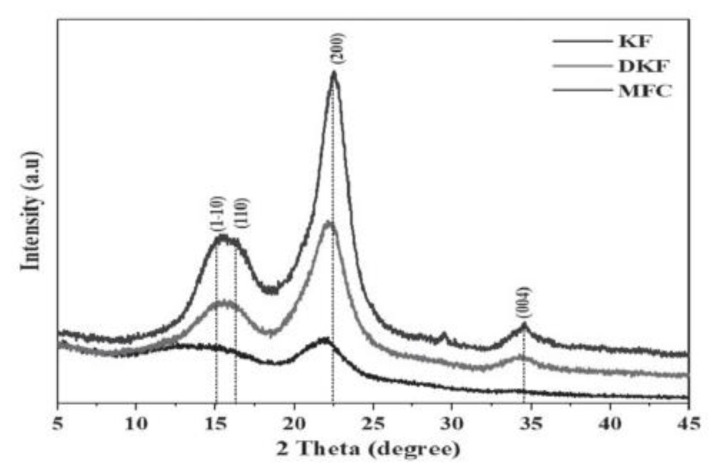
XRD pattern of KF, DKF and MFC [90].

**Figure 7:**
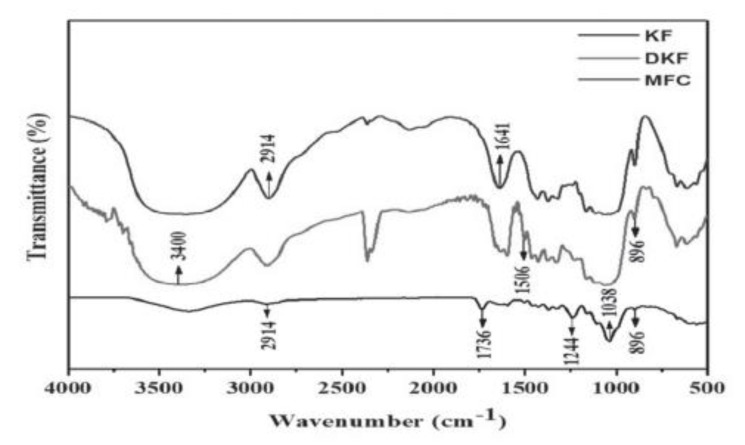
FT-IR spectra of KF, DKF and MFC [90].

In the study of Sartika et al. [90], KF was treated with NaOH and H_2_O_2_ to prepare delignified kapok fiber (DKF) and microfiber cellulose (MFC), respectively. Raw KF had flat surfaces that were still coated with wax, lignin, and hemicellulose in SEM images. Alkaline treatment caused the KF to become tangled and eroded, whilst acid treatment reduced the fiber diameter and destroyed the surface [90]. These changes in the structure of KF resulted from the removal of hemicellulose, the rearrangement of lignin [91], and the hydrolysis of cellulose [92] during pretreatment. Based on Figure 6, results of XRD analysis identified peaks (2θ) at 15°, 16°, 22°, and 34° that represented the crystallographic fields of (1–10), (110), (200), and (004), respectively. The peaks are attributed to the cellulose structure of KF [85,90]. FTIR analysis (Figure 7) showed the changes in composition and chemical structure of KF for each pre-treatment. The band at 3400 cm^−1^ refers to the OH stretching in cellulose, hemicellulose, and lignin [90]. The absorption band at 2914 cm^−1^ shows the C-H stretching vibration where the intensity increased after KF has undergone alkali and acid pre-treatment [90,93]. The bands at 1736 cm^−1^ and 1038 cm^−1^ indicate the C=O stretching of the lignin and hemicellulose and C-H stretching of the aromatic ring of lignin, respectively. After treatment with NaOH and H_2_O_2_, the peak intensity at 1736 cm^−1^ was observed to reduce significantly. Sartika et al. [90] concluded that alkali and acid-pre-treatments degrade and reduce the hemicellulose and lignin in KF. Meanwhile, glycosidic bonds of cellulose appeared at around 896 cm^−1^, which refers to the C-O-C [90,94].

In the study by Wang et al. [70], the KF surface was modified by acid and alkali treatment at the conditions of pH 7.0 and 80 °C and resulted in an adsorption capacity of 23.4 mg/g for the removal of lead. SEM images of KF under alkaline pre-treatment revealed that the hydroxyl and methoxy present in the interpenetrating polymer network can adsorb heavy metal ions and dyes because of its fishnet structure. Furthermore, the FTIR peaks at 3400 cm^−1^ show the characteristics of the hydroxyl (-OH) groups, which are directly connected to the adsorption capacity via chelating bonds that form between the fiber surface and lead ions [70].

When compared to raw KF, treated KF has a much higher adsorption capacity based on the adsorption conditions of pH 6.0, room temperature and an initial lead concentration of 80 mg/L. The adsorption capacity for the removal of lead in aqueous solution using untreated kapok fibers was determined to be 4.70 mg/g. This is a significantly low value when compared to the acid/alkali-treated kapok fiber, which has a value of 23.4 mg/g. The low adsorption capacity for lead, using raw KF, can be correlated to the existence of plant wax, which makes the fiber hydrophobic [95]. The application of acid/alkali treatment removes the plant wax, exposing functional groups such as hydroxyl, which has been linked to the adsorptive removal of heavy metal ions [70]. Furthermore, the change in surface properties increases the surface area of the fiber, potentially trapping more ions.

#### 3.1.2. Kapok Fiber Treated with Fenton Reaction

The Fenton reaction (Equation (1)) uses a combination of hydrogen peroxide and a ferrous or ferric salt that reacts with cellulose and lignin [96]. For the degradation of organic substrates, this method either depolymerizes or polymerizes the cellulose and lignin of fibers under acidic conditions (pH > 4) [97,98] whereby the reaction occurs between hydrogen on organic material and the hydroxyl radical (Equation (2)). Hydroxyl radicals can damage organic molecules (RH) such as lignin and cellulose, releasing water and carbon dioxide as a result (Equation (3)).

**Figure 8:**
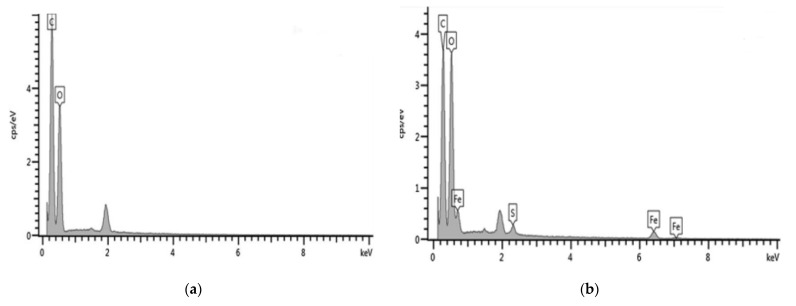
EDS analysis of (**a**) untreated KF and (**b**) modified KF using Fenton reaction [73]. Reprinted by permission from Springer Nature Customer Service Centre GmbH: Springer Nature, Environmental Earth Sciences, Removal of lead (II) from aqueous stream by hydrophilic modified kapok fiber using the Fenton reaction, Wang, D., Kim, D., Shin, C.H., Zhao, Y., Park, J.S., & Ryu, M., Copyright (2018).

**Figure 9:**
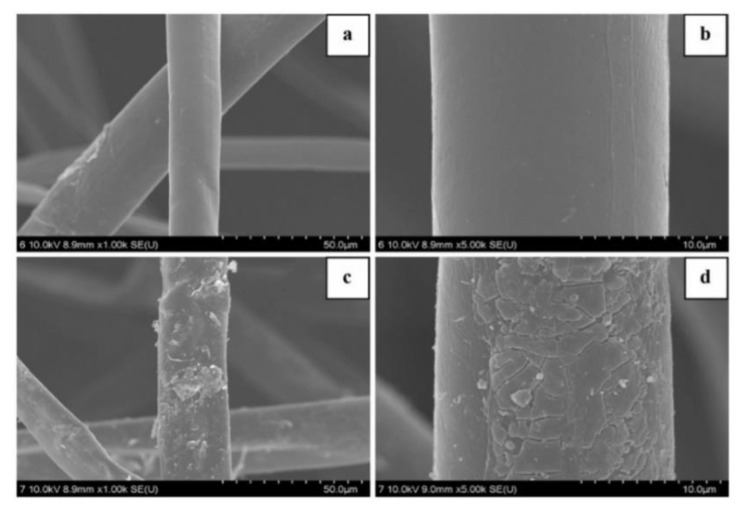
SEM micrographs at 10× and 50× magnification, respectively, of (**a**,**b**) raw KF and (**c**,**d**) KF under Fenton reaction [73]. Reprinted by permission from Springer Nature Customer Service Centre GmbH: Springer Nature, Environmental Earth Sciences, Removal of lead (II) from aqueous stream by hydrophilic modified kapok fiber using the Fenton reaction, Wang, D., Kim, D., Shin, C.H., Zhao, Y., Park, J.S., & Ryu, M., Copyright (2018).

Wang et al. fabricated a hydrophilic KF for lead removal via Fenton reaction (FRKF) using FeSO_4_ and H_2_O_2_. The elemental composition and distinctive peaks of untreated and modified KF are shown in EDS (energy dispersive x-ray spectroscopy) analysis (Figure 8). The primary elements in raw KF are carbon and oxygen. Sulfur and iron were present after the Fenton reaction. For raw KF, the FTIR peaks at 3343 cm^−1^ and 2917 cm^−1^ were due to the OH group and bond of CH_2_ and CH_3_, respectively. These bands are attributed to the presence of surface wax [50,59]. Meanwhile, the presence of lignin and xylan are observed at peaks 1737 cm^−1^, 1371 cm^−1^, and 1239 cm^−1^ [99]. Weak bands are also observed at 1595 cm^−1^, 1504 cm^−1^, and 1425 cm^−1^, which are related to the aromatic C–O in lignin [100]. The peak at 1035 cm^−1^ is attributed to the C–O–C stretching because of the existence of cellulose, hemicellulose, and lignin [101]. An increase in band intensity was detected after Fenton treatment, indicating that the surface of KF had been altered by the decomposition of lignin, wax, pectin, and natural oils, exposing new functional groups such as hydroxyl groups on the KF surface. SEM micrographs further confirms the decomposition of lignin, wax, and pectin because of the changes in surface morphology from a smooth to rough KF surface [73]. The rough surface topography (Figure 9c,d) is attributed to the removal of lignin, pectin, waxy compounds, and natural oils that give KF a smooth surface (Figure 9a,b). In addition, the hydroxyl radicals of the Fenton reaction could have oxidized the hydroxyl groups to become carboxyl groups, which in turn could have contributed to the increase in peak intensity.

This process successfully modified the surface structure of KF, which makes it suitable for heavy metal adsorption [73]. The adsorption capacity of the Fenton reaction treated fibers are higher with an adsorption capacity of 94.41 mg/g for the removal of Pb than the raw kapok fibers, which had an adsorption capacity of 4.70 mg/g and the acid/alkali treatment, which had 23.4 mg/g for its adsorption capacity. The comparison was based on the adsorption conditions of pH 6.0 and 25 °C.

Fe^2+^ + H_2_O_2_→ → → → Fe^3+^ + OH^−^ → + OH^•^ (1)

OH • RH→ → → → R• → + H_2_O (2)

R• + → RH → → → → CO_2_ → + H_2_O (3)

#### 3.1.3. Kapok Fiber Treated with Oxidation

Oxidation pre-treatment uses oxidizing agents such as ozone, peroxides, or chlorine-based solutions that decompose the lignin and emulsifies the hemicellulose in KF [102,103,104]. KF becomes porous and rougher on its surface through blended oxidation of chlorite and periodate that removes lignin and modifies the polysaccharides present [1,105]. This method also transformed the wettability of KF from hydrophobic to hydrophilic [106].

In the study of Wang et al. [107], KF was treated with a mixture of NaClO_2_ and glacial acetic acid prior to mercury ion removal from the aqueous system. Oxidized KF can be an excellent adsorbent for the removal of a targeted pollutant. The surface morphology of the treated KF exhibited a rougher surface, indicating that surface wax was removed [104,107]. This alteration in morphology was due to the bleaching and grafting properties of NaClO_2_ [72].

**Figure 10:**
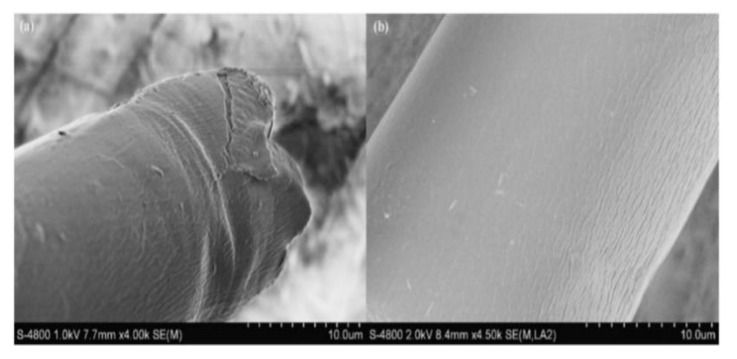
SEM images of (**a**) raw and (**b**) sodium chlorite treated KF [52]. Reprinted from Chemical Engineering Journal, 184, Liu, Y., Wang, J., Zheng, Y., & Wang, A., Adsorption of methylene blue by kapok fiber treated by sodium chlorite optimized with response surface methodology, 248–255, Copyright (2012), with permission from Elsevier.

The study by Liu et al. [52] showed that the surface of KF treated with sodium chlorite (NaClO_2_) transformed from hydrophobic to hydrophilic and was applied in the adsorption of methylene blue. This is because NaClO_2_ would generate chlorine dioxide and cause the oxidation of lignin. FTIR identified the peak at 3397 cm^−1^ as OH in cellulose, which became broader after treatment, indicating that lignin bonds were broken. There were no changes observed for a peak at 1643 cm^−1^ after treatment. The peaks attributed to lignin started to disappear at 1592 cm^−1^, 1504 cm^−1^, and 1463 cm^−1^. At 831 cm^−1^, the lignin of KF was completely broken [52] that resulted in the good hydrophilic property of treated KF [52,106]. The surface morphology of raw and treated KF is shown in Figure 10. Raw KF displayed a tube-like shape (Figure 10a) while no damage of the tube structure and surface of KF [104] was observed after pre-treatment (Figure 10b).

Wolok et al. [108] performed an oxidation pre-treatment on KF using sodium hypochlorite (NaClO) and NaClO_2_ that removes wax, natural oil, pectin, lignin, and hemicellulose. The increased hydrophilic properties of treated KF can be utilized for polymer application. FTIR results of untreated KF showed the presence of cellulose and surface wax at peaks 3342 cm^−1^ and 2916 cm^−1^, respectively [108,109]. A mixture of NaClO_2_ and NaOH pre-treatment decreases the peak intensity at 2916 cm^−1^ due to the oxidation of NaClO_2_ solution [75,108,110]. Another decrease in the intensity at peak 1732 cm^−1^ was observed, which implies the breakdown of lignin and hemicellulose [108,111]. The peak at 3342 cm^−1^ decreased with the treatment of the NaClO and NaOH solution due to damaged lignin [1,108]. Peaks at 1239 cm^−1^, 1041 cm^−1^, and 898 cm^−1^ indicated damage having occurred as a result of using the NaClO solution, specifically in lignin, polysaccharides, and cellulose, respectively [108,109,112]. Based on SEM analysis, the form and surface area of KF after oxidation pre-treatment using various solutions became wrinkled and rough. Particulates were also formed [108] during oxidation. Due to oxidation treatment, the loss of lignin, pectin, wax, and natural oil that covers the surface led to the improvement of the wettability and mechanical properties of KF [85,113].

The adsorption capacity obtained by NaClO_2_ treated fiber for the removal of methylene blue is 110.13 mg/g. The kinetic study indicates chemisorption as the rate-determining step in the removal of methylene blue using NaClO_2_ treated fibers. An increase in temperature affects the chemisorption mechanism as it increases the rate at which bonds form between the adsorbate and KF surface [58,62]. FTIR results show the peak at 831 cm^−1^ confirms the total disappearance of the lignin component of the fiber where most of the plant wax is located. As a result, the aqueous solution and the treated KF surface are able to make total contact. In an acidic solution, methyl blue is a cationic dye with a strong affinity to the fiber surface that would result in high adsorption capacity of the NaClO_2_ treated fibers [52].

### 3.2. Surface Modification of Kapok Fibers

#### 3.2.1. Polyaniline and Kapok Fiber

**Figure 11:**
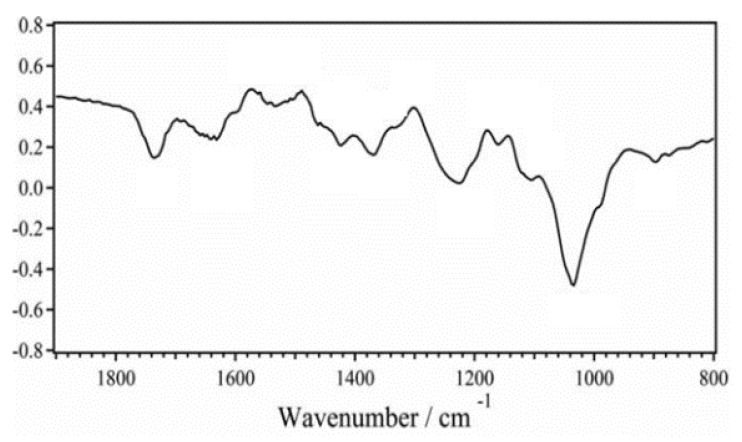
FTIR graph of polyaniline coated KF [28]. Reprinted from Water Science and Technology volume 78, issue number 5, pages 1137–1147, with permission from the copyright holders, IWA Publishing.

Herrera et al. [28] used an in situ oxidative polymerization technique to coat KF with polyaniline (PANI) for methyl orange and copper adsorption. KF was first treated with a combination of NaClO_2_ and glacial acetic acid before being coated with aniline monomer. The KF was also treated with an oxidant solution, a combination of ammonium persulfate (APS) and HCl. Before treatment, raw KF is hydrophobic with a water contact angle of around 120°. After surface modification, the PANI-coated KF reached complete wettability at a 0° water contact angle [28]. SEM images showed uncoated KF exhibited a microtubular structure with a middle lumen. After polymerization, a coarser surface was observed with a coating thickness of approximately 2.38 μm on the KF surface [28]. Figure 11 illustrates the C=O stretching and O-H bending of raw KF, which corresponds to the peaks at 1740 cm^−1^ and 1632 cm^−1^, respectively. Meanwhile, the presence of quinoid and benzenoid ring-stretching of PANI was found at peaks 1578 cm^−1^ and 1489 cm^−1^, respectively. Amine group is also detected at 1300 cm^−1^ peak. Furthermore, imine groups were found at 1404 cm^−1^ and 1146 cm^−1^ peaks. Additionally, various vibrations of carbohydrate present in KF were exhibited at peaks 1420 cm^−1^, 1366 cm^−1^, 1231 cm^−1^, 1038 cm^−1^, and 899 cm^−1^ [28,114].

The adsorption capacity of PANI-coated KF is 75.76 mg/g and 81.04 mg/g for the removal of methyl orange and copper, respectively. An increased adsorption capacity is observable and was attributed to PANI, as it is known to have numerous functional groups such as imine and amine groups that act as binding sites for the adsorption of heavy metal ions and dyes [28]. The PANI-coated KF of the present study provided high adsorption capacity for methyl orange and copper removal. The FTIR peaks at around 1578 cm^−1^ and 1489 cm^−1^ indicate that quinoid and benzenoid rings are present. In the molecular structure of PANI, the nitrogen atom of the imine group beside the quinoid ring is assumed to be reactive and may likely become a binding site for MO and Cu.

**Figure 12:**
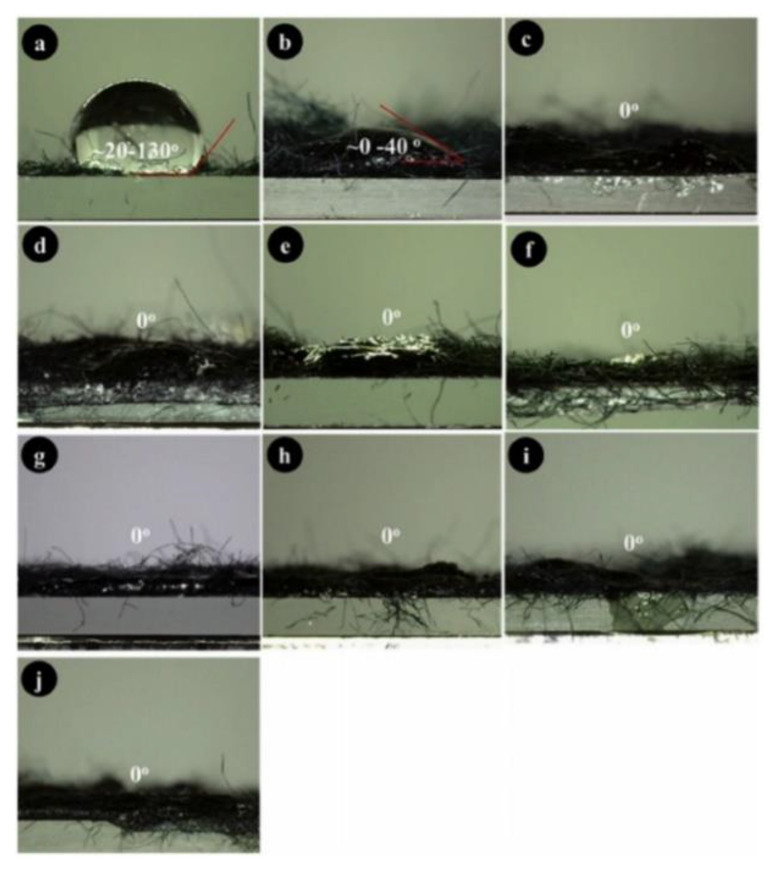
Water contact angle of PANI-KF nanocomposite with different ratio APS/aniline of (**a**) 0.2, (**b**) 0.4, (**c**) 0.6, (**d**) 0.8, (**e**) 1.0, (**f**) 1.2, (**g**) 1.4, (**h**) 1,6, (**i**) 1.8, (**j**) 2.0 [68]. Reprinted from Materials Chemistry and Physics, 243, Gapusan, R.B., & Balela, M.D.L., Adsorption of anionic methyl orange dye and lead (II) heavy metal ion by polyaniline-kapok fiber nanocomposite, 122,682, Copyright (2020), with permission from Elsevier.

Gapusan and Balela [68] fabricated a polyaniline (PANI)-KF nanocomposite by in situ polymerization in combination with NaClO_2_ pre-treatment for the removal of heavy metals and ionic dyes. Coating or immersing the fiber in a mixture of monomers and solvents is known as in situ polymerization [68,115]. To coat the NaClO_2_ pre-treated KF, a mixture of aniline monomers with ammonium persulfate (APS) was utilized. Figure 12 shows the measured water contact angles on PANI-coated KF under varying the ratio of APS and aniline. With a 0.2 to 0.4 ratio, it was observed that the contact angles were not uniform. These values range from 20° to 130° and 0° to 40° using the 0.2 and 0.4 APS/aniline ratio, respectively. This implies that the surface of the PANI-KF nanocomposite improved its hydrophilicity. Meanwhile, SEM images show that when the ratio of APS/aniline is at 0.2 to 0.4, a thin, uniform PANI layer was formed on the surface of KF. The average diameter of PANI-KF nanocomposite was observed to increase from 15.5 µm to 18.8 µm when the ratio of APS/aniline was also increased from 0.2 to 0.6. At ratios of 0.8 to 1.6, a thick polyaniline layer was observed on the surface layer of KF. At 1.4 ratio, the average diameter of PANI-KF nanocomposite was 23.6 μm. Meanwhile, a thinner polyaniline layer was recorded for the 1.8 and 2.0 ratio, with an average diameter decrease of around 19.8 µm to 22.2 µm. This is the effect of the over-oxidation of NaClO_2_ pre-treatment, as presented in the magnified SEM images [68].

The adsorption performance of PANI-KF nanocomposite fibers is determined to be 136.75 mg/g. The value is higher than that of the PANI-coated KF prepared by Herrera et al. [28], which had an adsorption capacity of 75.76 mg/g for the removal of MO. Both studies used the same pre-treatment and sorption methods [28,68]. However, the study of Gapusan and Balela [68] used a more controlled method of depositing PANI onto the surface of kapok. Moreover, adding cationic surfactants such as APS improves the adsorption capacity of composite for removing anionic contaminants such as MO. Hydrogen bonds are formed between the hydroxyl groups and the aniline amine groups during oxidative polymerization. On the KF surface, hydrogen bonding aids in the formation of a continuous and constant PANI layer. A higher concentration of APS applied has caused an increase in the number of PANI molecules deposited on the surface of KF, resulting in an increase in the amount of adsorbed MO. The more PANI molecules that are deposited on the surface of KF, more adsorption sites for heavy metal ions and dyes become available.

**Figure 13:**
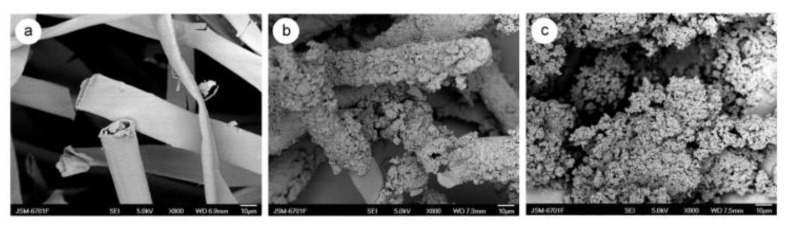
SEM images of (**a**) KF, (**b**) KF with polyaniline, and (**c**) polyaniline [71]. Reprinted (adapted) with permission from Zheng, Y., Liu, Y., & Wang, A., Kapok fiber oriented polyaniline for removal of sulfonated dyes. Industrial & engineering chemistry research, 51 (30), 10079–10087, Copyright 2012 American Chemical Society.

Zheng et al. [71] designed a PANI-oriented KF by the facile polymerization of aniline and chemical oxidation method that effectively removed sulfonated dyes such as Congo Red, Orange II, and Orange G. The synthesis of KF composite is performed by chemical oxidation through the mixing of both aniline monomer and APS for 30 min. The raw KF is then added into the mixture and would undergo a 16 h polymerization. SEM images (Figure 13) showed that the outer surface of KF is smooth and silky. Upon coating, a high concentration of aggregated rough particles of PANI was found on the surface of KF. The FTIR analysis shows a similar trend between PANI and PANI oriented KF, except that the adsorption bands of KF with polyaniline increased. The most notable adsorption bands for PANI oriented KF were 1741 cm^−1^ (carbonyl group in ester bond), 1244 cm^−1^ (amine stretching), and 1056 cm^−1^ (carbonyl group in carbohydrate region). These absorption bands both overlapped for KF and polyaniline, concluding that KF has combined successfully with polyaniline [71]. Response surface methodology (RSM) was performed to determine the optimum amount of KF and APS that would provide the highest adsorption capacity for Congo red, Orange-II, and orange G. The BET surface area of PANI-oriented KF was 21.80 m^2^/g when the ratio of the amount of KF and ammonium persulfate was 0.40/4.0 g.

The adsorption capacity of PANI-oriented KF was 40.82 mg/g, 188.7 mg/g, and 192.3 mg/g for Congo Red, Orange II, and Orange G, respectively. The treated KF has rougher surfaces, which increases the surface area so that the dyes may bind, thereby increasing the adsorption capacity. FTIR data reveal that the existing imine and amine groups are active adsorption species [44,71].

#### 3.2.2. Polyacrylonitrile-Coated KF

Agcaoili et al. [8] coated KF with polyacrylonitrile (PAN) using the surfactant-assisted method and its application in the removal of MO and copper. Before immersing KF in cetyltrimethylammonium bromide (CTAB) solution, impurities were removed with diluted ethanol. To catalyze the polymerization, potassium peroxydisulfate, or KPS, was also added. KF is known to have a highly hydrophobic surface, with a contact angle of 133° [8]. Upon PAN coating and after increasing the amount of CTAB, there was a decrease in the water contact angle within range from 12.15° to 0°, indicating that the KF composite has become a hydrophilic material. SEM images display large PAN particles with 4–12 μm in size on the surface of KF using 10 mg of CTAB. With 15 mg of CTAB, the particle size of coated PAN was approximately 2 μm, which was consistently distributed throughout the surface of KF. The homogeneous coating of PAN with a particle size of 0.67 μm was observed by applying 20 mg of CTAB onto the KF surface. Thus, coating KF using the surfactant-assisted method effectively distributed and arranged the polymer particles without damaging its surface area [8,115].

FTIR spectra of both raw and composite KF with different concentrations (10–40 mg) of CTAB exhibited C-H stretching, C-H bending, and C-C stretching at peaks 2900 cm^−1^, 1200 to 1400 cm^−1^, and 1020 cm^−1^, respectively. These peaks are mostly observed in cellulosic materials. At 3400 cm^−1^, the O-H stretching band of cellulose decreased after coating. PAN-coated KF showed C≡N peaks or the nitrile functional group at 2250 cm^−1^. These peaks are related to the successful coating of polyacrylonitrile to the KF [8,69].

The adsorption capacity of PAN-coated KF was determined to be 34.72 mg/g and 90.09 mg/g for MO and copper removal, respectively. Using surfactants such as CTAB and KPS during fabrication may have contributed to the increased adsorption capacity. The surfactants increased wettability and aided in the uniform coating of PAN on the KF surface. An FTIR analysis confirmed the presence of a nitrile functional group that has lone pairs of electrons, which could serve as a polar functional group that could attract heavy metal and dye ions [8]. However, PAN tends to form agglomerates on the surface of kapok. It was observed that utilizing CTAB as a surfactant resulted in fewer agglomerates and helped in the stable and even coating of the fibers.

#### 3.2.3. KF with the Self-Polymerized Dopamine (DA)

Yang et al. [56] synthesized dopamine (DA) coated KF via self-polymerization treated with NaClO_2_, which was utilized in the adsorption of mercury (Hg) ions from aqueous solution. DA was dissolved in a tris–HCl buffer solution and mixed with raw KF for 24 h. Then, DA-coated KF was washed with deionized water and then dried. Lastly, DA-coated KF was treated with a NaClO_2_ solution for 30 min before being rinsed and dried with deionized water. XPS spectra show peaks at 284 eV, 400 eV, and 580 eV, which is attributed to carbon 1s, nitrogen 1s, and oxygen 1s, respectively. The peaks of DA-coated KF were greater than the peaks of natural KF due to the formation of DA layer during polymerization [56,116]. Rough and uneven surfaces of DA-coated KF were detected by SEM where wax structure on the surface of KF has been removed that converted KF surface into hydrophilic material [10,56].

The adsorption capacity exhibited by DA-coated KF for the removal of Hg was 235.7 mg/g, which is remarkably high. SEM images showed the adherence of DA to the surface of KF. DA is known for being an excellent adhesive polymer. It has functional groups such as amine, imine, and catechol that can stick to the lignocellulosic regions of KF. Moreover, XPS spectra show organic elements such as C 1s, N 1s, and O 1s that react with the amine and imine through the formation of non-covalent and covalent bonds. Therefore, these interactions further contributed to the increase in the adsorption capacity of DA-coated KF [10,56]. The adsorption capacity for the raw KF was 39.9 mg/g for the removal of Hg. It was also established that due to the smooth surface characteristic of untreated KF, the adsorption capacity can be limited to low values. Smooth surfaces have a limited adsorption capacity for heavy metals and dyes. However, the removal of the wax layer by chemical treatment and incorporating polydopamine resulted in a rougher KF surface. This indicates the presence of a greater surface area and more binding sites that the Hg ions can adhere to. Moreover, the formation of quinone structures in DA-coated KF, which react with amines, created an interpolymer structure for the entrapment of Hg ions. These pre-treatments and modifications allowed for the increased adsorption capacity of DA-coated KF [8,56,68].

#### 3.2.4. DTPA Modified Kapok Fiber

Duan et al. [117] chemically modified KF with diethylenetriamine penta-acetic acid or DTPA to remove metal ions, i.e., lead, cadmium, and copper from aqueous solution. NaOH treatment was applied to obtain a hydrophilic KF. Then, the treated KF was added to the mixture of DTPA anhydride and dimethylformamide at 75 °C for 20 hrs. This process allows complete coating of DTPA to the KF. Results of SEM show that raw KF exhibits a cylindrical, hollow tube structure with open ends. After modification, KF displayed a wrinkled surface and the hollow structure collapsed. SEM images displayed that the fiber shrinks as its thickness increases from 0.7 to 2 µm [117]. The wrinkling and roughening of its surface imply an increase in surface area for better adsorption. The FTIR analysis shows the successful introduction of DTPA to the surface of KF. The FTIR analysis shows the disappearance of peak 1740 cm^−1^ (C=O group) after NaOH-treated KF, which is due to the removed lignin after treatment. However, the 1740 cm^−1^ band appears in the spectrum of DTPA-modified KF, which indicates that the ester and carboxyl are introduced after DTPA has been introduced. No changes were observed at the absorption bands 3410 cm^−1^, 2900 cm^−1^, and 1060 cm^−1^ for all samples, which means that some compositions of KF remain after surface modification. The presence of C=O stretching of hemicellulose is also noted at bands 1659 cm^−1^ and 897 cm^−1^ in NaOH-treated KF spectra [69,117]. The adsorption capacity of the DTPA modified KF for the removal of Cu, Pb and Cd are 101.0 mg/g, 310.6 mg/g, and 163.7 mg/g, respectively. The high adsorption capacity could be attributed to the addition of DTPA, which is a chelating agent that surrounds metal ions and form stable complexes [117].

## 4. Summary

### 4.1. Comparative Analysis on Results from Different Characterizations of KF Composites

FTIR spectra from various studies on pre-treatments show identical peaks. FTIR spectra of acid and alkaline-pretreated KF displayed a decrease in intensity. Acid pre-treatment removes the hemicellulose and lignin, which gives the KF its hydrophobicity. On the other hand, applying concentrated alkali solution to KF initiates de-esterification, whereby esters linked to the aromatic ring of lignin are removed. Likewise, the intensity in FTIR spectra of the Fenton reaction was observed to decrease. This allows for the conversion of KF from hydrophobic to hydrophilic, which enhances heavy metal ion adsorption capacity when hydroxyl, carboxyl, or aldehyde groups are added during the Fenton reaction. Meanwhile, oxidation pre-treatment caused an increased in intensity of peaks. Oxidizing agents produce chlorine dioxide during pre-treatment. This activates the oxidation reaction that eliminates the lignin present in KF. At band 1038 cm^−1^, the presence of lignin and hemicellulose linkage appeared after applying oxidation pre-treatment and Fenton reaction. Additionally, FTIR results displayed similar peaks in various surface modification studies. Hydroxyl stretching disappeared when KF was coated with PANI and PAN, since both used HCl to pre-treat the KF [28,71]. The presence of lignin for DTPA and PAN-based KF was not detected since both studies used NaOH pre-treatment. Thus, a lack of these functional groups means more amorphous sites are formed to allow for selective adsorption.

XRD spectra of raw, NaOH-treated, H_2_O_2_-treated and oxidized KF were assessed. At 2θ = 15–16°, peaks are present for oxidized KF. A peak at 2θ = 22° is observed in NaOH-treated and H_2_O_2_-treated KF. Lastly, 2θ = 34° was observed in NaOH-treated KF. These observations indicate that KF composites, i.e., composites based on a polymer or polymer blend, are favorable for the selective adsorption of heavy metals from an aqueous system due to the aggregating crystalline phase after pre-treatment.

Meanwhile, XPS peaks of C 1s, N 1s, and O 1s of DA-coated KF increased when compared to the raw KF. This is due to the development of the DA layer on the KF surface during self-polymerization. In addition, these elements are responsible for the structural rearrangement of the KF composite by forming an inter-polymer and intra-polymer network, which is responsible for entrapping the heavy metal ions.

Raw KF is hydrophobic due to the presence of wax that contains cellulose and lignin, which repel water molecules [28,115]. Additionally, using surfactants such as CTAB and APS during polymerization helped to convert a hydrophobic KF into a hydrophilic one. Varying the concentrations controls the thickness of polymer coating and distribution along the surface of KF [115].

Most of the SEM images after pre-treatment and surface modification displayed a rough surface morphology. The SEM images for oxidation treatment were observed to have a rough and wrinkled surface, which favors adsorption as it increases surface area. PAN-oriented KF were observed to have aggregated rough particles on the surface of KF. These aggregated particles may have imine and amine groups, which are active sites for adsorption. Moreover, PAN aggregates also contribute to the unevenness of the KF fibers which in turn contribute to better adsorption performance. The same is observed with PAN-coated kapok, which exhibited a rough morphology resulting in a larger surface area for more favorable adsorption. DA-coated KF has been observed to have an uneven and rough coating on the surface of KF. Meanwhile, SEM images for the DTPA coated KF was observed to display a wrinkled surface and collapsed structure.

### 4.2. Adsorption Capacity of Various KF Composites

Generally, the different adsorption behaviors reflected in the previous studies rely on several key factors that endowed them with unique adsorption capacities. Materials used, preparation techniques, and additives can affect the adsorption performance of the KF composites. In addition, operating parameters such as pH, adsorbent weight, and contact time are several factors that affect the adsorption performance [27].

The synthesis of FRKF is a promising pretreatment method because of the ability of hydroxyl groups to attack the organic material. In doing so, wax, lignin, and cellulose are removed, thereby changing the wettability of the fiber. Moreover, it also changed the surface morphology of the fiber from a smooth to rougher surface [73].

KF composites undergone chemical oxidation resulting in a modified fiber surface of the KF, which provided a low adsorption capacity. Under acidic conditions, NaClO_2_ produces ClO_2_ that contributes to the oxidation of lignin. This leads to lignin being destroyed and the formation of more amorphous sites occurred, which are sites for adsorption. However, low adsorption is caused by the lone attraction of ions to the chemically treated KF. Additionally, no addition of additives applied in the synthesis that could have further improve the adsorption performance of the fibers [52].

The study on PAN-coated KF was unique due to the use of CTAB, which assisted in polymer arrangement and deposition. CTAB assists with the negatively charged acrylonitrile monomer by initially creating a hydrophobic-hydrophobic interaction with the KF surface with positively charged heads directed outside [8,28].

Previous studies on PANI-coated KF and PANI-oriented KF showed that using PANI in its emeraldine salt state is advantageous due to the positively charged polaron and bipolaron sites succeeding the protonation of nitrogen atoms. PANI composites are used to remove basic, acidic, and neutral compounds such as dyes and heavy metals from aqueous solution. Additionally, all previous studies that utilized PANI used APS, which has a positive influence on the adsorption properties because APS serves as an initiator in the formation of PANI chains [8,68].

In the previous study involving the DA-Coated KF, it was determined that a quinone structure was formed after oxidation with catechol groups that reacted with amines. In turn, the formation of an inter and intra polymer network, such as a fishnet entrapping the contaminants occurred. This is also served as a unique additive as the polymer forms structures that specifically serve as a trapping site for the unwanted contaminant [56].

For DTPA modified KF, the synthesis of the fiber involved cutting the fiber into an average length of 50–100 µm by using a commercial juice extractor. This significantly contributed towards the increase in surface area of the fibers before subjecting it to pre-treatment with NaOH [117].

Overall, modified KF demonstrates different behaviors based on its treatment and polymer matrix that affect its overall adsorption capacity. The adsorption capacity of a KF composite material is dependent on several factors such as functional groups, surface chemistry, surface morphology, specific surface areas, pore diameter, and point of zero charges. Unfortunately, the previous studies that have been discussed did not measure some of the adsorbent characteristics, such as point of zero charge, pore diameter, specific surface area and surface chemistry.

### 4.3. Heavy Metal and Dye Adsorption Kinetic Study

The adsorption kinetics of heavy metals and dyes into KF is essential for selecting the best operating conditions for the full-scale batch process. Based on the literature survey, the heavy metal ions are charged solutes and hydrophilic in nature, whereas dyes are organic compounds that can be cationic, anionic or neutral. Thus, the pseudo-second-order model and Langmuir isotherm model were fitted and applied for batch adsorption. The pseudo-second-order model has a reliable performance in illustrating the kinetic data of KF composites. Conditionally, these kinetic models assume all the samples have a homogeneous surface, and the monolayer sorption arises on the surface of the KF composite. Meanwhile, the Langmuir isotherm model assumes the KF composite, where the adsorption occurs, has binding sites with identical energy levels and definite localized sites. If the kinetic data fit well with the pseudo-second order model, it implies that chemisorption is the rate-determining step of the adsorption system, whereby the surface of the adsorbent shares electrons with the contaminants and creates a strong covalent bond. Additionally, chemisorption has high enthalpy that occurs at different temperatures. In chemisorption, the adsorbent has a high affinity for the adsorbate, which could be either a heavy metal ion species or dye molecules, bounded by complex kinetic processes. Results of the isotherm study show that the Langmuir model best describes the adsorption system [118,119,120,121,122,123,124].

Table 1 shows the different adsorbents and their corresponding adsorption capacities. The table also shows that the kinetic studies for all the composites follow a pseudo-second-order equation, which means chemisorption is the rate-determining step regardless of the type of composite material used as an adsorbent. Raw and modified KF and its adsorptive removal follow the Langmuir isotherm, which implies that monolayer adsorption occurs for both heavy metals and dyes on the adsorbent material. It has been observed that almost all the adsorption capacity values were much higher in cases where the adsorbent underwent pre-treatment and surface modification. The study of Yang et al. (2020) showed that the adsorption capacity of Hg can be significantly improved via coating KF with DA. The Hg adsorption capacity of 39.9 mg/g of raw KF was observed to increase to 235.70 mg/g upon coating KF with DA [56]. The same results are observed in the study of Wang et al. (2014), which demonstrated a low adsorption capacity of 4.70 mg/g for unmodified KF. Treatment of KF with NaOH resulted in a higher adsorption capacity of 23.4 mg/g in the removal of Pb from aqueous solution [70]. Coating KF with DPTA resulted in an excellent adsorption capacity for Pb, Cu and Cd of 101.0, 310.6, and 163.7 mg/g, respectively [117]. This implies that an improvement in the adsorption capacity of various contaminants can be attained by the application of chemical pre-treatment or by coating KF with polymers and chelating agents.

## 5. Conclusions

The present work provides a summary of the various pre-treatment and surface modifications that have performed on KF and its modified forms in the removal of dyes and heavy metals from synthetic solutions. KF is recognized for its hydrophobic-oleophilic property, which limits its application as an oil-adsorbing material. In general, natural KF has poor selectivity towards dyes and heavy metals in aqueous solutions. To overcome this limitation, studies on physical and chemical treatment as well as coated KF have been reviewed. The conversion of the KF surface from hydrophobic to hydrophilic is achieved via acid and alkali treatment, oxidation treatment and Fenton reaction treatment. These methods can remove some of the plant wax, natural oils, pectin and lignin on the KF surface. The pre-treatment techniques remove the waxy layer of KF, which improves the wettability of the fibers and exposes highly functional groups such as hydroxyl and methoxy. These groups serve as binding sites and are correlated with the adsorption performance of the fibers. In addition, the pre-treated fibers or the KF undergoing surface modification exhibit altered surfaces. The pre-treatment application such as Fenton reaction and coating KF with DA, polyaniline, and acrylonitrile resulted in aggregated particles on the KF surface with the presence of grooves.

Overall, pre-treatment methods and the coating of KF with polymers lead to an improved overall adsorption capacity. The DTPA-modified KF exhibited an excellent adsorption capacity in the removal of Cu, Cd and Pb, while KF treated with Fenton or NaOH showed a significant increase in Pb adsorption. In addition, KF coated with DA also displayed an improved adsorption capacity for Pb. Factors such as functional groups, surface chemistry, and surface morphology affect the overall adsorption performance of modified KF. Determining the surface area and average pore diameter of the modified KF would help in examining the characteristics of modified KF and its removal of various contaminants from synthetic solutions in future studies.

## Figures and Tables

**Table 1:** Various adsorption capacity of heavy metals and dyes using KF and KF composites.

Composite	Adsorbate	Operational Parameters	Adsorption Capacity (mg/g)	Isotherm	Kinetic	Reference
Raw KF	Hg	pH = 8.0; 0.1 g adsorbent; volume = 100 mL	39.9	Langmuir	Pseudo-second order	[56,70]
	Pb	pH = 4.5; 0.1 g adsorbent; volume = 100 mL	4.70			
Alkali-treated KF	Pb	pH = 4.5; 0.1 g adsorbent; volume = 100 mL	23.4	Langmuir	Pseudo-second order	[70]
Fenton Reaction	Pb	pH = 6.0; 0.1 g adsorbent; volume = 30 mL; temperature = 25 °C	94.41	Langmuir	Pseudo-second order	[73]
NaClO_2_ treated KF	Methyl Blue	pH = 6.0; 0.05 g adsorbent; volume = 25 mL; temperature = 30 °C	110.13	Langmuir	Pseudo-second order	[52]
PANI-coated KF	Cu	pH = 4.3; 0.03 g adsorbent; volume = 20 mL; temperature = 25 °C	81.04	Langmuir	Pseudo-second order	[28]
	Methyl Orange	pH = 6.5; 0.03 g adsorbent; volume = 20 mL; temperature = 25 °C	75.76			
PANI-oriented KF	Congo Red	pH = natural pH; 0.025 g adsorbent; volume = 25 mL; temperature = 25 °C	40.82	Langmuir	Pseudo-second order	[71]
	Orange II		188.7			
	Orange G		192.3			
PANI-nanocomposite	Methyl Orange	pH = 6.0; 0.04 g adsorbent; volume = 50 mL; temperature = 25 °C	136.75	Langmuir	Pseudo-second order	[68]
	Pb	pH = 6.0; 0.04 g adsorbent; volume = 50 mL; temperature = 25 °C	63.60			
PAN-coated KF	Cu	pH = not given; 0.03 g adsorbent; volume = 20 mL; temperature = 25 °C	90.09	Langmuir	Pseudo-second order	[8]
	Methyl Orange		34.72			
DA-coated KF	Hg	pH = 8.0; 0.1 g adsorbent; volume = 100 mL	235.7	Langmuir	Pseudo-second order	[56]
DTPA modified KF	Cu	pH = 4.5; 1.0 g adsorbent; volume = 1000 mL; temperature = 25 °C	101.0	Langmuir	Pseudo-second order	[117]
	Pb	pH = 4.5; 0.8 g adsorbent; volume = 1000 mL; temperature = 25 °C	310.6			
	Cd	pH = 4.5; 1.0 g adsorbent; volume = 1000 mL; temperature = 25 °C	163.7			

## Data Availability

Not applicable.
